# A pilot observation using ultrasonography and vowel articulation to investigate the influence of suspected obstructive sleep apnea on upper airway

**DOI:** 10.1038/s41598-024-56159-2

**Published:** 2024-03-13

**Authors:** Shumit Saha, Anand Rattansingh, Rosemary Martino, Keerthana Viswanathan, Anamika Saha, Nasim Montazeri Ghahjaverestan, Azadeh Yadollahi

**Affiliations:** 1 Department of Biomedical Data Science, School of Applied Computational Sciences, Meharry Medical College, Nashville, TN USA; 2 KITE-Toronto Rehabilitation Institute, University Health Network, Toronto, ON Canada; 3 Institute of Biomedical Engineering, University of Toronto, Toronto, ON Canada; 4 Institute of Health Policy, Management, and Evaluation, Dalla Lana School of Public Health, University of Toronto, Toronto, ON Canada; 5 Toronto General Hospital, University Health Network, Toronto, ON Canada; 6 Krembil Research Institute, University Health Network, Toronto, ON Canada; 7 Department of Speech-Language Pathology, University of Toronto, Toronto, ON Canada; 8 Rehabilitation Sciences Institute, University of Toronto, Toronto, ON Canada; 9 Department of Otolaryngology – Head & Neck Surgery, University of Toronto, Toronto, ON Canada; 10 Department of Electrical and Computer Engineering, Smith′s Engineering, Queen′s University, Kingston, Canada

**Keywords:** Respiratory tract diseases, Machine learning, Statistical methods

## Abstract

Failure to employ suitable measures before administering full anesthesia to patients with obstructive sleep apnea (OSA) who are undergoing surgery may lead to developing complications after surgery. Therefore, it is very important to screen OSA before performing a surgery, which is currently done by subjective questionnaires such as STOP-Bang, Berlin scores. These questionnaires have 10–36% specificity in detecting sleep apnea, along with no information given on anatomy of upper airway, which is important for intubation. To address these challenges, we performed a pilot study to understand the utility of ultrasonography and vowel articulation in screening OSA. Our objective was to investigate the influence of OSA risk factors in vowel articulation through ultrasonography and acoustic features analysis. To accomplish this, we recruited 18 individuals with no risk of OSA and 13 individuals with high risk of OSA and asked them to utter vowels, such as /a/ (as in “Sah”), /e/ (as in “See”). An expert ultra-sonographer measured the parasagittal anterior–posterior (PAP) and transverse diameter of the upper airway. From the recorded vowel sounds, we extracted 106 features, including power, pitch, formant, and Mel frequency cepstral coefficients (MFCC). We analyzed the variation of the PAP diameters and vowel features from "See: /i/" to "Sah /a/" between control and OSA groups by two-way repeated measures ANOVA. We found that, there was a variation of upper airway diameter from “See” to “Sah” was significantly smaller in OSA group than control group (OSA: ∆12.8 ± 5.3 mm vs. control: ∆22.5 ± 3.9 mm OSA, p < 0.01). Moreover, we found several vowel features showed the exact same or opposite trend as PAP diameter variation, which led us to build a machine learning model to estimate PAP diameter from vowel features. We found a correlation coefficient of 0.75 between the estimated and measured PAP diameter after applying four estimation models and combining their output with a random forest model, which showed the feasibility of using acoustic features of vowel sounds to monitor upper airway diameter. Overall, this study has proven the concept that ultrasonography and vowel sounds analysis may be useful as an easily accessible imaging tool of upper airway.

## Introduction

Obstructive sleep apnea (OSA) affects 10% of the adult population and is characterized by repetitive collapse of the upper airway during sleep^1^. The gold standard assessment for OSA requires participants to spend the night in the sleep laboratory and undergo polysomnography with up to 20 sensors attached to different parts of the body^2^. Due to the complex nature of polysomnography and its high cost, 80% of individuals with OSA are not diagnosed^3^. Undiagnosed and untreated OSA is a major risk factor for developing heart disease, hypertension, and stroke^4^. Furthermore, in a preoperative setting, failure to employ suitable measures before administering full anesthesia to patients with OSA who are undergoing surgery may lead to developing complications after surgery^5^. Currently, screening of OSA before performing surgery is achieved by questionnaires such as STOP-Bang, Berlin, or Epworth sleepiness score^6,7^. However, these questionnaires are subjective in nature, which leads to a low (10–36%) specificity in detecting sleep apnea^6,8^. Moreover, these questionnaires do not address anomalies of the anatomy of the upper airway, which makes intubation difficult^9^. Developing accessible and user-friendly technologies for the imaging of the upper airway would lead to a meaningful and accurate screening of OSA during wakefulness.

Magnetic resonance imaging (MRI) and Computerized Tomography (CT) studies have been used to assess the upper airway dimensions during wakefulness^10–13^. These studies have shown that the upper airway is typically narrower in individuals with OSA than in healthy individuals^10–13^. However, MRI or CT is expensive and not easily accessible in certain settings, such as in the emergency department, or before surgery. Additionally, the use of CT increases exposure to the neck structures to ionizing radiation, particularly to the thyroid, with potentially detrimental effects^14^. By comparison, ultrasound imaging is far more accessible in such situations, is less expensive, and does not use ionizing radiation. For these reasons, point of care ultrasound (PoCUS) systems are becoming established in assessing individuals with certain applications such as pulmonary^15^, diaphragmatic^16^, and hemodynamic instability^17^. PoCUS is also gaining rapid attention for assessing the anatomical landmarks of the upper airway and its association with OSA^18–21^. In line with this research, a previous study from our group has shown the reliability and validity of the ultrasonographic measurement of the upper airway dimension during normal breathing in individuals with a high risk of OSA^22^.

Several studies have shown that there is a significant overlap in the size of the upper airway during wakefulness between individuals with OSA and healthy controls^23–25^. This could be due to the activation of upper airway muscle tone during wakefulness^26^. To address this problem and to better understand the pathogenesis of OSA, controlled maneuvers to simulate different levels of upper airway narrowing that may occur during sleep could be considered^27^. Vowel articulation is a great example of a controlled maneuver that changes tongue position in the oral cavity and could simulate upper airway narrowing. During the articulation of the frontal vowel (i.e. "/i/: See"), the tongue moves forward, and consequently, the upper airway widens^28^. On the contrary, the tongue moves backward and narrows the upper airway during back vowels (i.e. "/a/: Sah", "/e/: Set") articulation^28^. Furthermore, previous studies have explored the utility of speech articulation in screening individuals with OSA^29–32^. Robb et al. have shown that the formant frequencies of the vowels "/i/" and "/a/" are lower in individuals with OSA than in the healthy controls^29^. Moreover, several studies have used the features of speech in screening individuals with OSA^30–32^. These studies have shown that speech articulation may be used as a potential tool in screening individuals with OSA. However, it is not clear whether vowel articulation could reflect variations in the upper airway dimension between individuals with and without OSA, and what are the potential underlying reasons behind these acoustic features that can differentiate individuals with and without OSA.

The current study was conducted to investigate the changes in the upper airway dimension during vowel articulation in people with and without risk of OSA. Two sets of modalities were used to investigate: (a) ultrasonographic, and (b) acoustic features of vowel sounds. This study was structured into three sections. In the first section, we used ultrasonography to measure the upper airway dimension during vowel articulation. We investigated the variation in the upper airway dimension between vowels in those with and without risk of OSA. In the second section, we evaluated the effect of OSA on acoustic features of vowel sounds and assessed the relationship between acoustic features and ultrasonography-based upper airway dimension. Finally, in the third section, we implemented machine learning models to estimate the ultrasonography-based upper airway dimension from the acoustic features of vowel sounds.

## Methods

### Study participants

Adults older than 18 years of age were recruited. There were no exclusion criteria based on sex or body mass index (BMI).

### Data measurement

#### Upper airway dimension measurement during vowel articulation

We used ultrasonography to measure the parasagittal anterior–posterior (PAP) diameter of the upper airway during vowel articulation^22^. Canon ultrasound machine Aplio i700 (Canon Medical, Tochigi, Japan) was used to assess the PAP diameter. A curved transducer probe (1–6 MHz) was used for the measurement.

**Figure 1:**
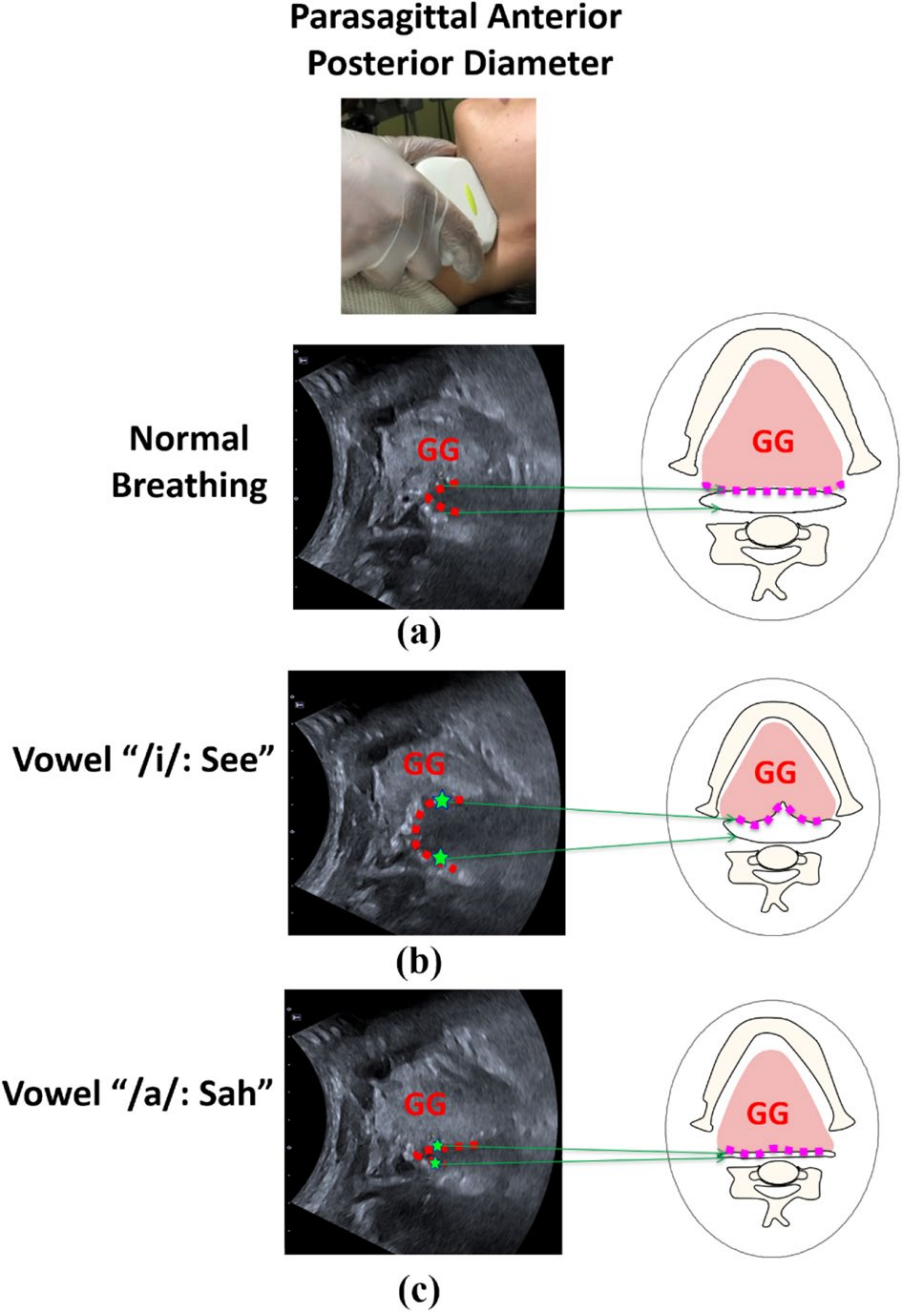
(**a**) Parasagittal anterior posterior (PAP) diameter measurement: The transducer probe is placed in a submandibular lateral oblique position, with its superior margin abutting the angle of the left mandible. This reveals the lateral oblique view in the submandibular region. The dotted red line shows the shape of the upper airway. (**b**) During vowel “See”, the upper airway widens as shown by the dotted red line. (**c**) However, during vowel “Sah”, the upper airway narrows. The distance between two starts is the PAP diameter. The schematic diagram shows the corresponding region and upper airway of the ultrasonography image.

To measure the PAP diameter, the transducer probe was placed in a submandibular lateral oblique position, with its superior margin abutting the angle of the left mandible. In this position, the airway appeared as a curved inverted-U shaped echogenic line during normal breathing (Fig. 1a)^20,22^. In this curve-shaped view, one part of the curve showed the reflection from the base of the tongue/genioglossal muscle, which was the anterior part of the upper airway. The other part of the curve showed the posterior part of the upper airway. Figure 1b shows the appearance of the upper airway in the vowel "See: /i/". The hyperechoic lines were widened, which indicated more opening of the upper airway. Two stars in the image showed the anterior and posterior border of the upper airway. The distance between the two stars was measured as the PAP diameter. On the contrary, in the vowel "Sah: /a/", the tongue moved backward, the upper airway was almost collapsed (Fig. 1c), and the measured PAP diameter was reduced.

The ultrasound examination was performed by an expert ultrasonographer with more than twenty years of experience. All the PAP diameter measurements were done manually by the technician who was blinded to the participants' demographics. For each vowel, we recorded the PAP diameters 5 times and averaged the 5 measurements to determine the final value.

### Recording of the vowel sounds

We asked the participant to articulate the vowels in the following order: /i/ as in “see”, /u/ as in “soo”, /a/ as in “sah”, /e/ as in “set”, and /o/ as in “so”. To record the vowels, a microphone was placed 12 cm above the participant's mouth. The 12 cm was maintained by a measuring tape, and the microphone (Sony ECM-44B) was fixed by using a tripod stand. The sound signals were digitized and saved to a memory card using sampling rates of 15,300 Hz. A sound meter was placed at 12 cm from the participant's mouth to make sure all the vowels were uttered at the same distance. We asked the participants to articulate their vowels in their usual way and style of speaking. There was no indication or instructions given to the participants while the utterance of the vowels on their loudness or pitch.

### Study protocol

First, we measured the participants' height and weight. Participants were asked to lie in a supine position without any head support (pillow). Neck circumference was measured at the level of cricothyroid cartilage using a measuring tape. Then, ultrasound examination was performed to measure the PAP diameter of the upper airway. During the examination, the participants were asked to remain still and articulate vowels on cue.

### Sleep disordered breathing assessment

"NoSAS" score was used to assess the risk of OSA^33^. NoSAS stands for Neck circumference, obesity, Snoring, Age, and Sex. NoSAS scores give 4 points for having a neck circumference ≥ 40 cm, 3 points for 25 ≤ BMI < 30 kg/m^2^ or 5 points for BMI ≥ 30 kg/m^2^, 2 points for reported snoring, 4 points for age ≥ 55 years, and 2 points for being male. Thus, NoSAS score ranges from 0 to 17. When the NoSAS score is ≥ 8, the individual has a high probability of OSA. This threshold of NoSAS score was validated on 2121 participants and showed higher accuracy than other questionnaires such as STOP-Bang and Berlin questionnaires^33^. A research coordinator who was blinded to the ultrasonography measurement performed the assessment.

### Data analysis

Analysis of the data is divided into 3 parts. In the first part, we extracted the vowel sound features. In the second part, we performed statistical analysis to understand the difference in PAP diameter and vowel sound features between the high-risk vs low-risk OSA groups. In the third part, we developed machine-learning models to estimate the PAP diameter from the vowel sound features.

### Part 1: vowel feature extraction

From the recorded sound signals, vowel segments were extracted manually by listening to the sounds. 'PRAAT', a computerized program for labeling audio data, was used to label and export the vowel segments^34^. Vowel segments were first downsampled to 10,000 Hz, and then 5th order Butterworth bandpass filtered between 100 and 3000 Hz to remove low and high-frequency noises.

For each vowel, 106 features were extracted (Table 1 in the supplementary file). The features included the pitch, formants, power-related and spectral magnitudes. We calculated the pitch frequency based on the robust algorithm for pitch tracking^35^. Additionally, for calculating three formants (F1, F2, and F3), vowels were pre-processed using a Hamming window (window size of 20 ms) and a pre-emphasizing filter. Then, the 8th order linear predictive coding (LPC) spectrum of the vowels was estimated to extract formants^36,37^.

Furthermore, we calculated the average power, relative power, and spectral centroid from the estimated power spectral density (PSD). We estimated the PSD based on the Welch method using a Hamming window of 20 ms (512 FFT points) and a 90% overlap between adjacent windows^38^. Both the average power and spectral centroid were calculated for the entire frequency band (100–3000 Hz). Also, average power, relative power, and spectral centroid were calculated for several sub-bands, including 100–500 Hz, 500–1000 Hz, 1000–1500 Hz, 1500–2000 Hz, 2000–2500 Hz, 2500–3000 Hz, 100–1000 Hz, 1000–2000 Hz and 2000–3000 Hz^39^.

Moreover, we extracted the mean and standard deviation of the Mel frequency cepstral coefficients (MFCC) for 13 bands^40^. MFCC was determined by the discrete cosine transform of a log power spectrum on a mel-scale of frequencies^40^. In addition, prominent features in speech processing such as Chroma energy^41^, spectral contrast^42^, spectral roll-off frequencies, and zero-crossing rate were extracted from vowel sounds. We used the "Librosa: a Python package for audio and music signal processing" for extracting these features^43^.

### Part 2: statistical analysis

The differences between the PAP diameters between vowels were analyzed by the one-way Analysis of Variance (ANOVA). The difference in PAP diameters and vowel sounds feature between two vowels in the same group were assessed by the paired *t*-test (for normally distributed data) or Wilcoxon rank test (for not normally distrusted data). To assess the difference in PAP diameters and the vowel sounds features between OSA and control group, we performed the independent *t*-test or Mann–Whitney *U* test based on normality. Furthermore, we analyzed the variation of the PAP diameters and vowel features from "See: /i/" to "Sah /a/" between control and OSA groups by two-way repeated measures ANOVA. Moreover, we performed a correlation analysis between the vowel sounds feature and the measured PAP diameters. We performed the Pearson or Spearman correlation based on the normality of the data.

Statistical analyses were performed by R (version 3.6.1) and two-tailed p < 0.05 was considered as significant. Data are presented as mean ± SD for normally distributed data and median and interquartile range for non-normally distributed data.

### Part 3: estimation of the ultrasound-based upper airway diameter from vowel sounds feature using machine learning models

**Figure 2:**
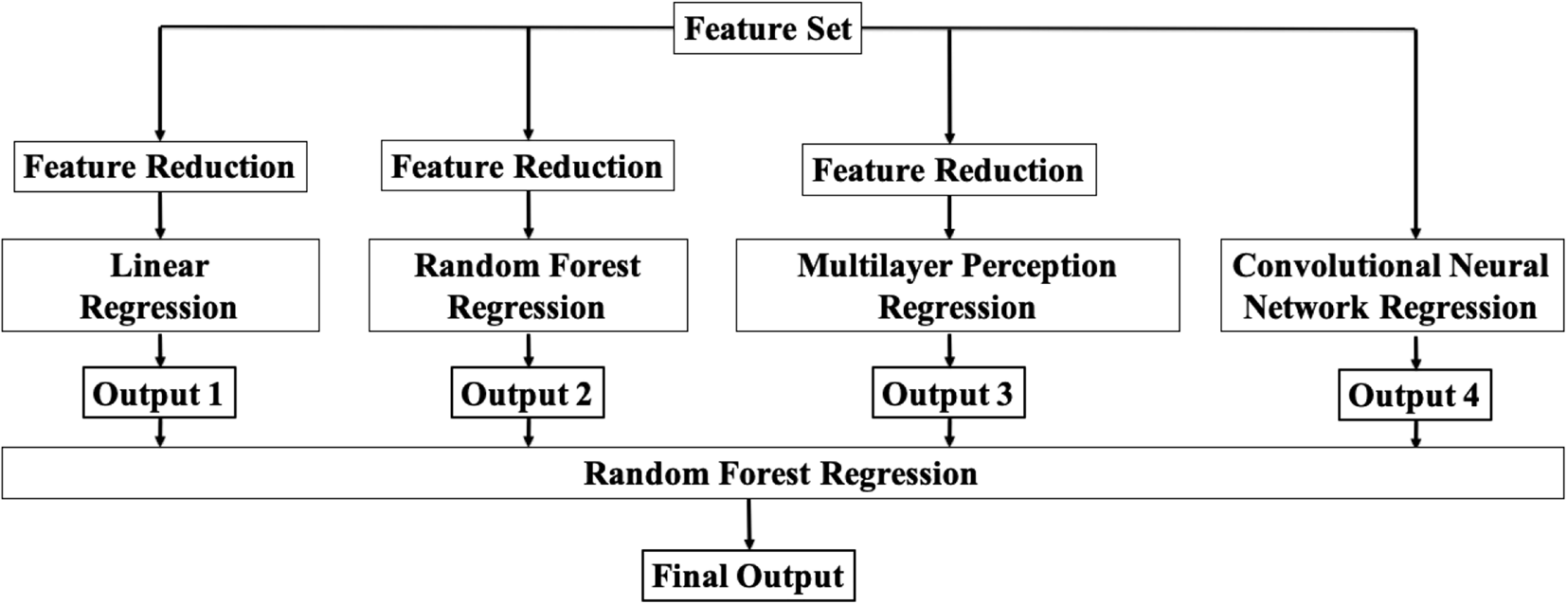
Algorithm flowchart for estimation of the parasagittal anterior–posterior (PAP) diameter from vowel sounds feature.

To estimate the PAP diameters from extracted vowel features, we used a six-fold cross-validation technique. In each iteration, we trained the data on five-folds and tested them on the remaining fold. For each fold, we used 125 vowel data points for training and 25 vowel data points for testing in each fold of the six-fold cross-validation. We used a two-step model to estimate the PAP diameters (Fig. 2). In step 1, we developed four different regression models. The models were (a) linear regression, (b) random forest regression, (c) artificial neural network regression, and (d) convolutional neural network regression. Thus, we obtained four outputs from the four models. In step 2, these four outputs were combined by a random forest regression to obtain the final output (Fig. 2). Detailed methodology and implementation of these models can be found in the Supplementary File (Sect. 2). After six-fold cross-validation, we obtained the output for all testing sets. We used root mean square error (RMSE) and correlation coefficient to evaluate the performance of our estimation algorithm.

### Ethical approval

The study was approved by the research ethics board of the University Health Network, Toronto, Canada. All participants provided written consent before participating in the study. All experiments were performed in accordance with relevant guidelines and regulations.

## Results

31 individuals participated in this study. Table 1 shows the demographics of participants, grouped into healthy (NoSAS < 8) and those with the risk of OSA (NoSAS ≥ 8). As NoSAS score categorized the high risk of OSA based on age, BMI, and neck circumference, these factors were significantly higher in individuals with a high risk of OSA compared to the healthy subjects.

**Table 1:** Demographics.

Characteristics	All subjects	Control (NoSAS < 8)	High risk of OSA (NoSAS ≥ 8)
Sex (M/F)	12/19	5/13	7/6
Age, years	43.22 ± 14.89	35.11 ± 11.97	54.54 ± 11.59*
BMI, kg/m^2^	28.32 ± 6.20	24.73 ± 3.74	33.29 ± 5.77*
Neck circumference, cm	36.68 ± 4.98	33.59 ± 5.64	34.21 ± 3.30*

*BMI* body mass index. *P < 0.05 between control subjects and those at high risk of sleep disordered breathing.

### Ultrasonographic measurements analysis

**Figure 3:**
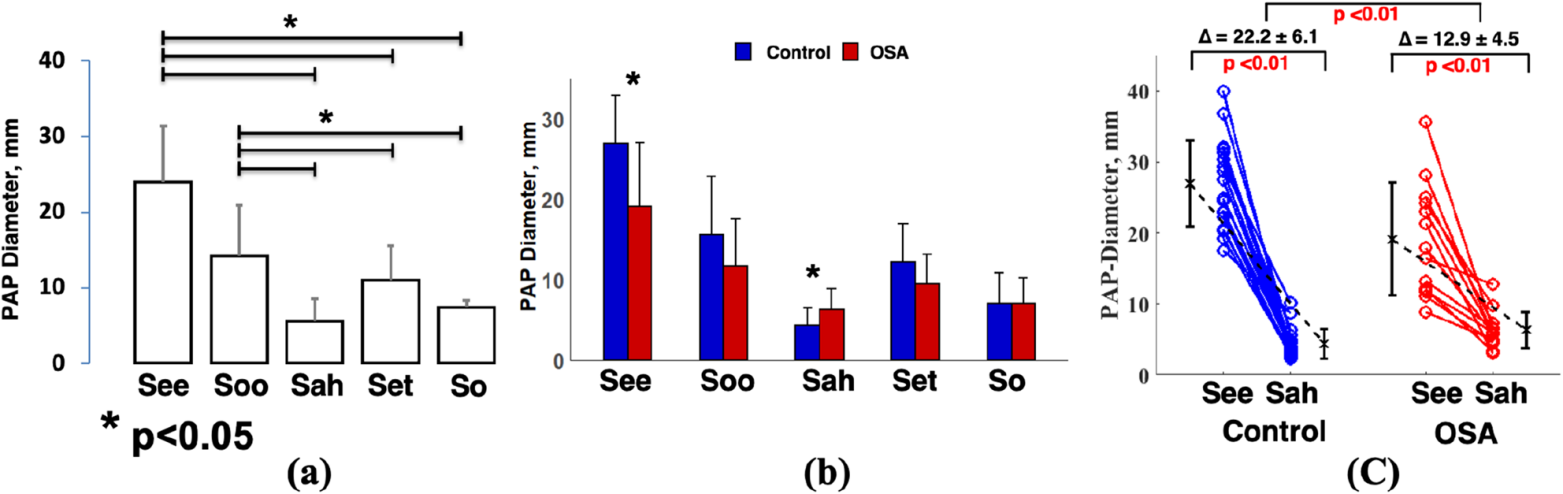
(**a**) The parasagittal anterior–posterior (PAP) diameters are smaller in back vowels (i.e. /a/, /e/ and /o/) than the frontal vowels (i.e. /i/, /u/). (**b**) PAP diameter in “See” is significantly smaller in high risk OSA group than control subjects. Furthermore, PAP diameter in “Sah” is significantly higher in high risk OSA than the control group. (**c**) The variation of PAP diameter from “See” to “Sah” is significantly lower in individuals with high risk of OSA than control.

The PAP diameters were significantly smaller in vowels “/a/: Sah”, “/e/: Set”, and “/o/: So” than the vowels “/i/: See” and “/u/: Soo” (Fig. 3a). We found that PAP diameters in the vowel “See” were significantly smaller in the OSA group than control group (control: 26.9 ± 6.0 mm vs OSA: 19.1 ± 7.9 mm, p < 0.05, Fig. 3b). Furthermore, PAP diameters in vowel “Sah” were significantly higher in OSA than control group (control: 4.3 ± 2.0 mm vs OSA: 6.3 ± 2.5 mm, p < 0.05, Fig. 3b). Also, the variation of upper airway diameter from “See” to “Sah” was significantly smaller in OSA group than control group (OSA: ∆12.8 ± 5.3 mm vs. control: ∆22.5 ± 3.9 mm OSA, p < 0.01, Fig. 3c).

### Acoustic analysis of the vowel sounds

#### Section 1: difference in vowel sound features between OSA vs control groups

**Figure 4:**
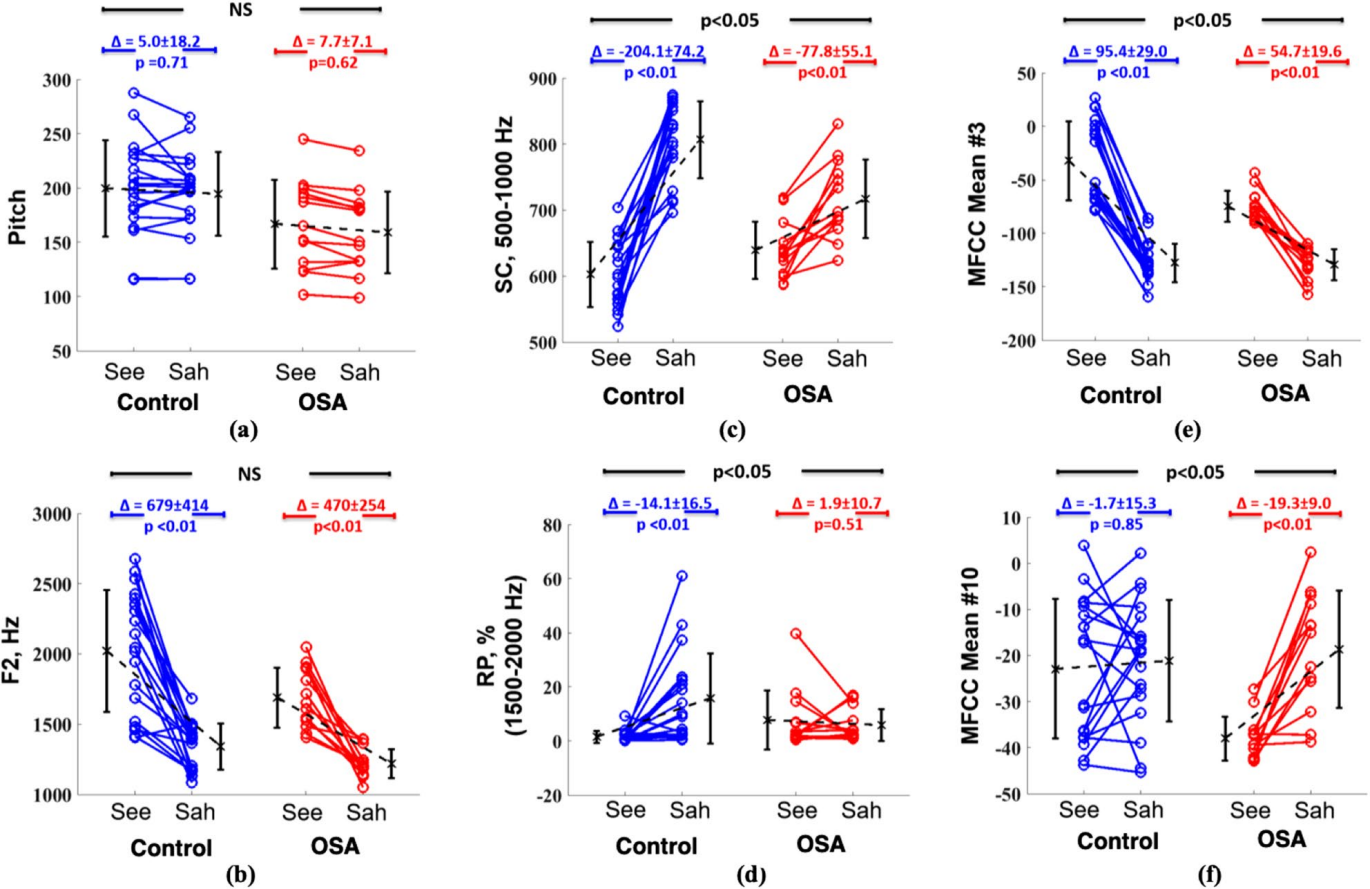
The variation of acoustic features of vowel sounds from “See” to “Sah” in control and OSA group: (**a**) Pitch, (**b**) Second formant (F2), (**c**) Spectral centroid in 500–1000 Hz, (**d**) Relative power in 1500–2000 Hz, (**e**) Mean of MFCC in band number 3, and (**f**) Mean of MFCC in band number 10 (*NS:* not significant).

The pitch frequencies were significantly lower in the OSA groups than in control in “See” (OSA: 166.8 ± 39.2 Hz vs. control: 199.7 ± 43.0 Hz OSA, p < 0.05) and “Sah” articulation (OSA: 159.2 ± 36.2 Hz vs. control: 194.7 ± 37.3 Hz, p < 0.01). However, there was no significant variation in the pitch frequency from “See” to “Sah” in OSA and control groups (OSA: ∆7.7 ± 7.71 Hz vs control: ∆5.0 ± 18.2 Hz, p > 0.05, Fig. 4a).

Similar to the pitch frequency, the 2nd formant (F2) was significantly lower in the OSA groups than in control in “See” (OSA: 1690.2 ± 204.2 Hz vs. control: 2021.7 ± 420.7 Hz, p < 0.05), and “Sah” articulation (OSA: 1219.1 ± 101.5 vs. control: 1243.1 ± 160.1, p < 0.01). However, the variation in F2 from “See” to “Sah” was not significantly different between control and OSA groups (control: ∆679 ± 414 Hz vs. OSA: ∆470 ± 254 Hz, p = 0.14, Fig. 4b).

The magnitude of variation in spectral centroid frequencies in 500–1000 Hz from “See” to “Sah” was significantly smaller in OSA group than control (OSA: ∆–77.8 ± 55.1 Hz vs. control: ∆–204.1 ± 74.2 Hz, p < 0.01, Fig. 4c). This finding was attributed to the fact that the spectral centroid was significantly higher in OSA group during “See” articulation (OSA: 639.4 ± 41.5 Hz vs. control: 602.8 ± 48.2 Hz, p < 0.01). On the contrary, during “Sah” articulation, the spectral centroid was significantly lower in the OSA group (OSA: 717.2 ± 56.7 Hz vs control: 807.0 ± 56.9 Hz, p < 0.01). Similar to the spectral centroid, changes in the relative power in 1500–2000 Hz from “See” to “Sah” was significantly smaller in the OSA group than control (OSA: ∆1.9 ± 10.7 Hz vs control: ∆–14.1 ± 16.5 Hz, p < 0.01, Fig. 4d). The MFCC in band 3 was significantly lower in OSA group during “See” articulation (OSA: − 74.0 ± 13.8 vs control: − 32.0 ± 36.1, p < 0.01), while there was no significant difference between groups during “Sah” articulation (OSA: − 129.4 ± 13.9 vs control: − 127.4 ± 17.5, p = 0.71). Changes in the mean of MFCC in band number 3 from “See” to “Sah” was significantly smaller in the OSA group than control (OSA: ∆54.7 ± 19.6 vs control: ∆95.4 ± 29.0, p < 0.01, Fig. 4-e). Moreover, the changes in the mean of MFCC in band 10 from “See” to “Sah” was significantly higher in the OSA group than control (OSA: ∆–19.3 ± 9.0 vs control: ∆–1.7 ± 15.3, p < 0.01, Fig. 4f). This finding was attributed to the fact that the MFCC in band 10 was significantly lower in OSA group during “See” articulation (OSA: − 37.9 ± 4.5 vs control: − 22.8 ± 14.7, p < 0.05), while there was no significant difference between the groups during “Sah” articulation (OSA: − 18.6 ± 12.2 vs control: − 21.0 ± 12.7, p = 0.59).

#### Section 2: correlation between vowel sounds and PAP diameters

**Figure 5:**
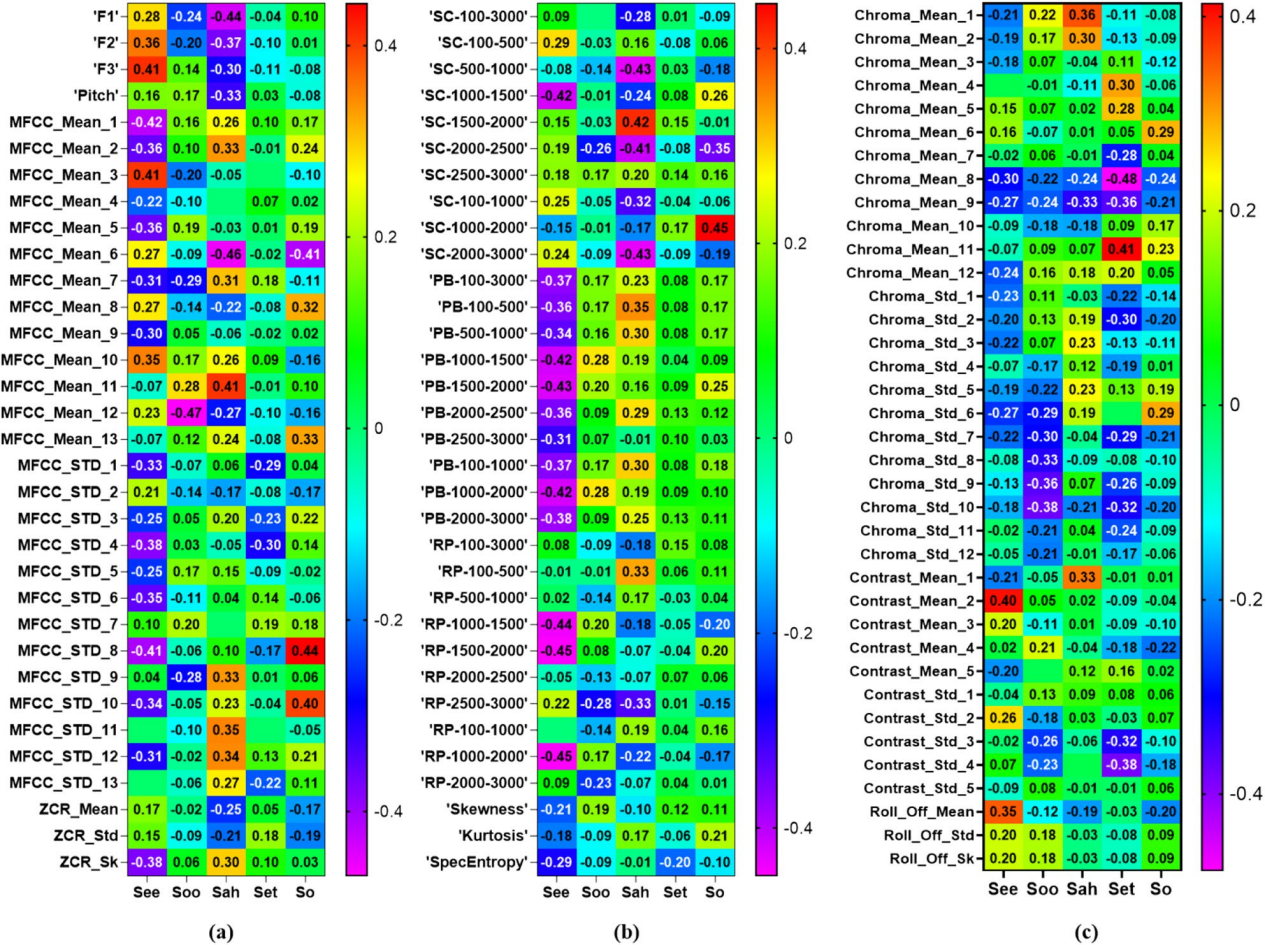
Heatmap of the correlation coefficients between the ultrasonography measured parasagittal anterior posterior (PAP) diameter  and acoustic features of vowel sounds: (**a**) pitch, formants (F1, F2, F3), mel frequency cepstrum coefficient (MFCC)–mean and standard deviation, and Zero crossing rate (ZCR), (**b**) spectral centroid (SC), band power (PB), relative power (RP) in 10 frequency bands, skewness, kurtosis, and entropy (SpecEntropy), (**c**) chroma, contrast, and roll-off frequency. (Created by GraphPad Prism: https://www.graphpad.com/).

In Fig. 5, we showed the heatmap of the correlation coefficients for all features of vowel sounds and the PAP diameters. There were significant correlations between decreases in the PAP diameters measured during “See” articulation and decreases in the F2 (r = 0.36, p = 0.04), F3 (r = 0.41, p = 0.02), and mean of MFCC in band 3 (r = 0.41, p = 0.02). Furthermore, decreases in the PAP diameters measured during “See” articulation were associated with the increases in the band power (r = − 0.42, p = 0.01), relative power (r = − 0.44, p = 0.01), and spectral centroid (r = − 0.42, p = 0.01) in the frequency range of 1000–1500 Hz. While articulating “Sah”, decreases in the PAP diameters were significantly associated with the increases in the F1 (r = − 0.44, p = 0.01) and spectral centroid in 500–1000 Hz (r = − 0.43, p = 0.01). These significant correlations show that the vowel sounds feature can be used to estimate the PAP diameter.

#### Section 3: estimation of the ultrasound-based upper airway diameter from vowel sounds features using machine learning models

To estimate the PAP diameter from the vowel sounds features, we developed four models in step-1 and combined the results of the four models in step-2. Table 2 shows the RMSE and the correlation coefficient (r) between ultrasound-based PAP diameter and estimated PAP diameter from vowels. Among the four models we developed in step 1, we obtained the highest correlation coefficient for the artificial neural network (r = 0.73, p < 0.001) model. In terms of higher correlation, the artificial neural network model was followed by the linear regression model (r = 0.72, p < 0.001), convolutional neural network model (r = 0.70, p < 0.001), and random forest model (r = 0.68, p < 0.001). After combining the output of these four models in step 2, we obtained the highest correlation coefficient (r = 0.75, p < 0.001) and the lowest RMSE (5.70 mm) (Table 2).

**Table 2:** Estimation of the PAP diameter from vowel sounds feature.

Testing dataset result					
Model phase	Model name	Fold wise RMSE, mm	Overall RMSE, mm	Fold wise testing correlation	Overall testing correlation, r
Phase 1	LR	5.82 ± 1.18	5.94	0.73 ± 0.12	0.72*
	RF	6.10 ± 1.13	6.20	0.72 ± 0.12	0.68*
	ANN	5.72 ± 1.11	5.83	0.74 ± 0.05	0.73*
	CNN	5.96 ± 1.40	6.12	0.67 ± 0.16	0.70*
Phase 2	RF	5.64 ± 0.74	5.07	0.76 ± 0.06	0.75*

*LR* linear regression, *RF* random forest regression, *ANN* artificial neural network regression, *CNN* convolutional neural network regression, *RMSE* root mean square error.

*p < 0.01.

## Discussion

The main novelty of our study was to show that vowel articulation in supine posture may provide important pathophysiological information about the upper airway dimension in individuals with a high risk of OSA. The most important findings of our study were: (1) variation of parasagittal anterior–posterior (PAP) diameter from “/i/: See” to “/a/: Sah” was lower in individuals with a higher risk of OSA compared to controls, (2) acoustic features of vowel sounds were significantly different between those with a high risk of OSA and control individuals, and were aligned with our ultrasonography findings; (3) vowel sounds features had significant associations with the ultrasonography based airway measurement; and (4) vowel sounds features can be used to estimate the upper airway dimension with high accuracy.

Our results showed that the variation in the PAP-diameter from the “See” to “Sah” was significantly lower in individuals with a high risk of OSA than control subjects. These results suggested that compared to the healthy controls, the tongue movement during articulating “See” to “Sah” was less in individuals with a high risk of OSA. Taken together, less variation in PAP diameters during articulating “See” and “Sah” may be influenced by the high volume, weight, and fat deposition of the tongue along with less tongue stiffness^13,44–46^. Future studies could validate the use of PAP measurement during vowel articulation as a tool to assess the anatomical and mechanical properties of tongue.

We further investigated the relationship between the measured PAP-diameters and acoustic features of vowel sounds, along with comparing the differences of these features between control and OSA groups. The shape of the tongue influences the formant frequencies, and less movements of the tongue would result in smaller variations in the formant frequencies between vowels^47^. Although we did not find significant variation in F2 from “See” to “Sah”, we observed a trend of smaller variation of F2 in the OSA group than the control. We found that F2 of “Sah” and “See” were smaller in OSA than in the control group. This result was in line with the previous studies, which showed that F2 of “See: /i/” was lower in the individuals with OSA than non-OSA groups^29,48^. These results were further supported by the association between lowering F2 and reduction in the PAP diameter during “See” articulation. These results suggested that while the variation of formant between vowels may not be used as an indicator of tongue movement, F2 of “See” may be an important feature for screening individuals with OSA.

Furthermore, we found that during the articulation of “See”, the vowel sounds were louder in the OSA group than controls, as assessed by the spectral centroid and relative power. However, during the “Sah” articulation, the vowel sounds intensity was lower in the control group. These results were in line with our findings from ultrasonography data. It shows that the intensity of the vowel sounds increased with more narrowing in the upper airway. These results may provide proof of concept for the potential application of the intensity of vowel sounds to assess the narrowing of the upper airway in individuals with OSA.

Moreover, we showed that the averages of MFCC bands were significantly different in vowel articulation in the OSA group, especially during “See” articulation. Our results suggested that the perception of the vowel sounds in the human auditory system was different for the OSA group than the control. Our results were further supported by previous studies, where an experienced speech pathologist could distinguish individuals with OSA by their speech articulation^49^. Furthermore, several studies have demonstrated the importance of MFCC features in classifying individuals with OSA^30,31^. Based on these results, the lower movement of the tongue during speech articulation in the OSA group could be one of the potential factors of these MFCC differences.

Finally, we showed that the vowel sounds feature could estimate the PAP diameter of the upper airway with low error and high correlation. Developing four estimation models and combining their output with a random forest model provided the highest accuracy. Therefore, our study provided the importance of developing multiple estimation models rather than one model. Our finding provided the proof of concept that the acoustic features of vowel sounds can be used as a simple and easy tool to assess the upper airway size. One importance of this finding is that these acoustic features could be extracted from snoring sounds during sleep. Therefore, analyzing these acoustic features from snoring may provide important insights into the upper airway dimension during sleep, which should be validated in future studies.

Overall, we demonstrated that the vowel articulation could be used as a useful intervention to simulate upper airway narrowing and can be used for differentiating those at high risk of OSA. To the best of our knowledge, this was the first study that employed vowel articulation during ultrasound-based upper airway assessment in OSA-risk individuals. Thus, vowel articulation could be used as a potential tool in the point of care ultrasonography. Furthermore, we showed that the less tongue movement and lower variation in the upper airway might be potential underlying links of the acoustic feature difference between OSA and control groups. This is the first study that presented the possible connection of the speech feature difference between individuals with and without OSA. Taken together, we demonstrated the utility of vowel articulation in evaluating the upper airway in individuals with a high risk of OSA.

Our study was subject to several limitations. We used the NoSAS score to screen the OSA. As NoSAS score may accurately identify individuals with severe OSA^33^, our results may be more applicable to the severe OSA population. Nonetheless, future studies are required to evaluate our results based on the gold-standard assessment of OSA with polysomnography. Furthermore, the positioning of the ultrasonography transducer probe is vital in the visualization of the upper airway. Although the ultrasound technique can be easily taught, the reproducibility of this study may require someone who has some experience in ultrasound imaging to reproduce this study. Additionally, we did not assess inter-operator variability with another sonologist. Moreover, it is noteworthy to mention that estimating lower formants with LPC may have inaccuracies. This inaccuracy may not have a bias in our estimation model as we did use formant as the main feature, nevertheless it should be addressed in future studies. Finally, our current dataset is limited in terms of sample size. Future studies are required to access our results in a larger population.

In conclusion, our study showed that the upper airway dimension variation in different vowel articulation might play an important factor in the OSA group. Both the ultrasonography and acoustic feature-based analysis supported our results. Therefore, vowel articulation could be used as a tool to evaluate the upper airway size, tongue posture, and ultimately to screen for individuals with OSA.

### Supplementary Information

Supplementary Information.

## Data Availability

The deidentified data will be available upon request. For request, please email azadeh.yadollahi@uhn.ca.
