# Supplementary material for: A pilot observation using ultrasonography and vowel articulation to investigate the influence of suspected obstructive sleep apnea on upper airway
Source: Sci Rep. 2024 Mar 13;14:6144. doi: 10.1038/s41598-024-56159-2 (PMC10937936; doi:10.1038/s41598-024-56159-2)
Supplement: Supplementary file 1 — Supplementary Information. [file 41598_2024_56159_MOESM1_ESM.docx]

**Supplementary File**

**Section 1: Feature Extraction from Vowel Sounds**

Table S1 shows the extracted feature set from the vowel sounds.

**Table S1:** Feature Set

| **Feature Name** | **# of Features** | **Feature Symbols** |
| --- | --- | --- |
| Pitch, Hz | 1 |  |
| Three Formants, Hz | 3 | F1, F2, F3 |
| Skewness | 1 | S_k_ |
| Kurtosis | 1 | K_u_ |
| Spectral Entropy | 2 | SE |
| Spectral Centroid | 12 | SC |
| Band Power | 10 | BP |
| Relative Power | 10 | RP |
| Chroma-Mean | 12 | C-M |
| Chroma-Standard Deviation | 12 | C-Std |
| Mel Frequency Cepstral Coefficient (MFCC) -Mean | 13 | Mfcc-M |
| Mel Frequency Cepstral Coefficient (MFCC)-Standard Deviation | 13 | Mfcc-STD |
| Contrast-Mean | 5 | Con-M |
| Contrast-Standard Deviation | 5 | Con-Std |
| Roll off frequency | 3 | RF |
| Zero Crossing Rate | 3 | ZCR |
| **Total Features per vowel** | **106** |  |

**Section 2: Estimation of the ultrasound based pharyngeal airway diameter from vowel sounds feature:**

We marked the vowel as follows: "See", "Soo", "Sah", "Set", and "So" as 1, 2, 3, 4, and 5, respectively. We used the extracted 106 features along with the markings of the vowels for the estimation of the PAP diameter. We used a six-fold cross-validation technique. In each fold, we trained the data on five-folds and tested them on the remaining fold. Thus, for each fold, we had 80% of the data for training and 20% of the data for testing.

We used a two-phase model to estimate the PAP diameter of the pharyngeal airway (Figure 2). In phase 1, we developed four different regression models. The models were a) linear regression, b) random forest regression, c) artificial neural network regression, and d) convolutional neural network regression. Therefore, we obtained four outputs from the four models. In phase 2, these four outputs combined by a random forest regression to obtain the final output (Figure 2).

*Phase 1 Models:*

*Linear Regression Model:* Before using the linear regression model, we reduced the vowel feature set. We used the principal component analysis (PCA) to reduce the feature set. We obtained six features from the PCA analysis. We used the linear regression with the six features to estimate the PAP diameter. To implement the model, we used the "LinearRegression" from "scikit learn" library [1].

*Random Forest Regression Model:* We used the six features that were extracted by PCA analysis. These six features were then used in the random forest regression model. We used 100 trees in the random forest algorithm. To implement the model, we used the "RandomForestRegressor" from "scikit learn" library [1].

*Artificial Neural Network Model:* To train the artificial neural network, we used 47 features. The selection of these 47 features were based on PCA analysis. The 47 features were standardized by removing the mean and scaling to the unit variance. Then, the standardized feature set was inputted in the input layer of the artificial neural network architecture (Figure S1). The network consisted of three hidden layers with 200, 100, and 25 neurons in the first, second, and third layer, respectively. We used the "logistic" activation function for the hidden layers. The limited memory Broyden–Fletcher–Goldfarb–Shanno algorithm (BFGS) was used for weight optimization. To implement the model, we used the "MLPRegressor" from "scikit learn" library [1].


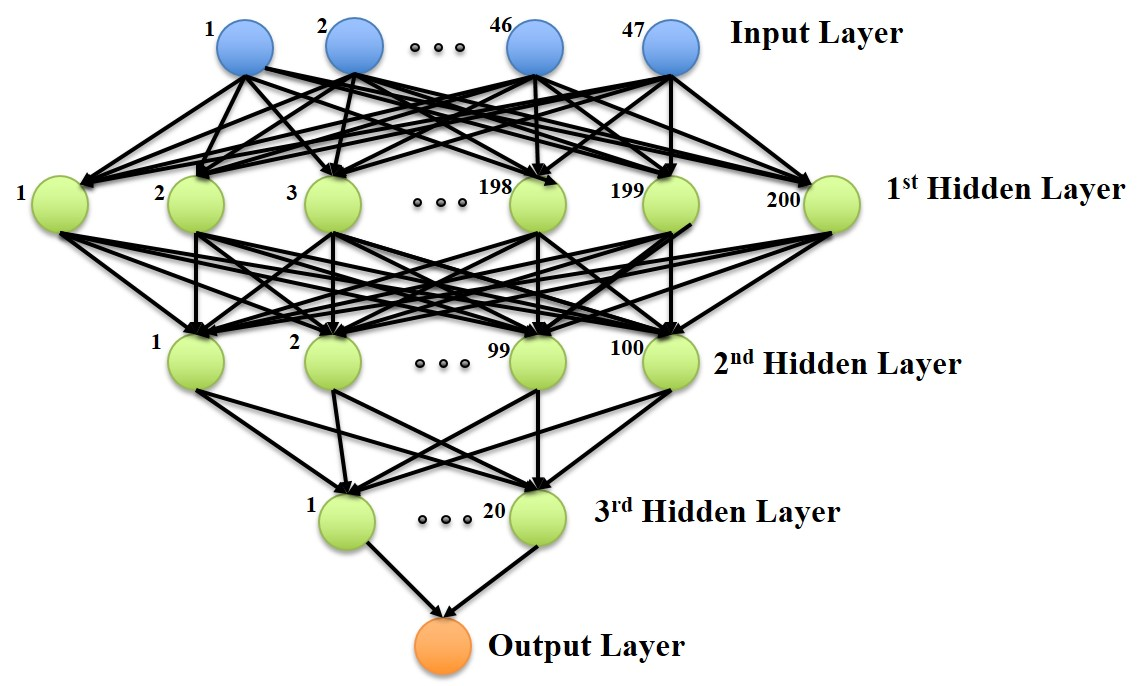


**Figure S1: ANN Architecture**

*Convolutional neural network (CNN) regression model*: In this model, we used all the 107 features. We first standardized all the features. We used five layers of 1-dimensional CNN. The kernel size of the five layers were 5, 5, 3, 2, and 2, respectively (Figure S2). The stride of one was used in all the layers. For the non-linearity, we used the ReLu activation function. To reduce the sensitivity of the output feature map, we introduced pooling layers to downsample the feature map with a kernel size of 2. The pooling layers were added after the first, second, third, and fifth CNN layers. To prevent overfitting, we used dropout with 0.5 and 0.2 on the outputs of first and second CNN layers. The values of the fifth CNN layers were flattened and then connected to a dense layer.


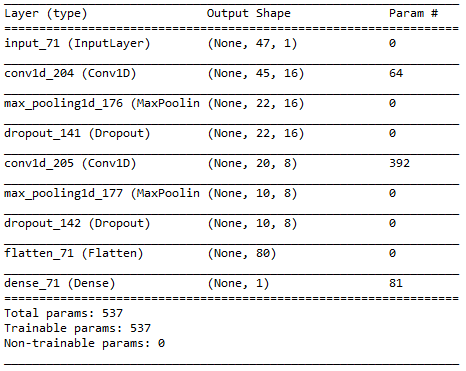


**Figure S2: CNN Regressor**

Network parameters were optimized using the 'mean squared error' loss function with the 'Adam’ optimizer. To prevent overfitting, ‘early stopping’ was used to monitor the loss. If the loss did not improve for 50 epochs, training was stopped. The regression model was implemented using Keras python deep learning library version 2.2.4 with TensorFlow GPU backend version 1.14.0 and CUDA 9.1.

*Phase 2 Model:*

*Random Forest Regression Model:* We used the four outputs of phase 1 models as the input of phase 2 random forest regression model. We used 100 trees in the random forest algorithm. The output of the phase 2 random forest model was considered as the final output.

After six-fold cross-validation, we obtained the output for all the testing set. We used root mean square error (RMSE) and correlation coefficient to evaluate the performance of our estimation algorithm.

**References**

[1] F. Pedregosa *et al.*, "Scikit-learn: Machine learning in Python," *the Journal of machine Learning research,* vol. 12, pp. 2825-2830, 2011.
